# Supplementary material for: Self-supervised learning for classifying paranasal anomalies in the maxillary sinus
Source: Int J Comput Assist Radiol Surg. 2024 Jun 8;19(9):1713–21. doi: 10.1007/s11548-024-03172-5 (PMC11365849; doi:10.1007/s11548-024-03172-5)
Supplement: Supplementary file 1 — (pdf 5469 KB) [file 11548_2024_3172_MOESM1_ESM.pdf]

Supplementary material: Self-supervised learning for classifying paranasal anomalies in the maxillary sinus

DEBAYAN BHATTACHARYA[1,2], FINN BEHRENDT[1], BENJAMIN TOBIAS BECKER[2], DIRK BEYERSDORFF[3], ELINA PETERSEN[4], MARVIN PETERSEN[5], BASTIAN CHENG[5], DENNIS EGGERT[2], CHRISTIAN BETZ[2], ANNA SOPHIE HOFFMANN[2], AND ALEXANDER SCHLAEFER[1]

[1] *Institute of Medical Technology and Intelligent Systems, Technische Universitaet Hamburg, Germany*
[2] *Department of Otorhinolaryngology, Head and Neck Surgery and Oncology*
[3] *Clinic and Polyclinic for Diagnostic and Interventional Radiology and Nuclear Medicine*
[4] *Population Health Research Department, University Heart and Vascular Center*
[5] *Clinic and Polyclinic for Neurology*
[1] *University Medical Center Hamburg-Eppendorf, Hamburg, Germany*

This supplemental document presents a formal definition of state-of-the-art self-supervised methods used in the main manuscript , accompanied by implementation details and illustrations. It also contains ablation study on the impact of reconstruction loss and post-processing used for the self-supervision task on the downstream classification task.

1. AUTOENCODER

Consider an encoder $E(.)$ and a symmetric decoder $D(.)$. Consider $x \in R^{64\times 64\times 64}$ is the maxillary sinus (MS) volume belonging to the unlabelled dataset D_u. A forward pass through the encoder and decoder signifies non-linear compression and decompression of x, respectively. Formally $\hat{x} = D(E(x))$. Notably, there are no skip connections between the encoder and decoder to prevent a trivial copying operation. Training employs the L1 reconstruction loss on D_u.

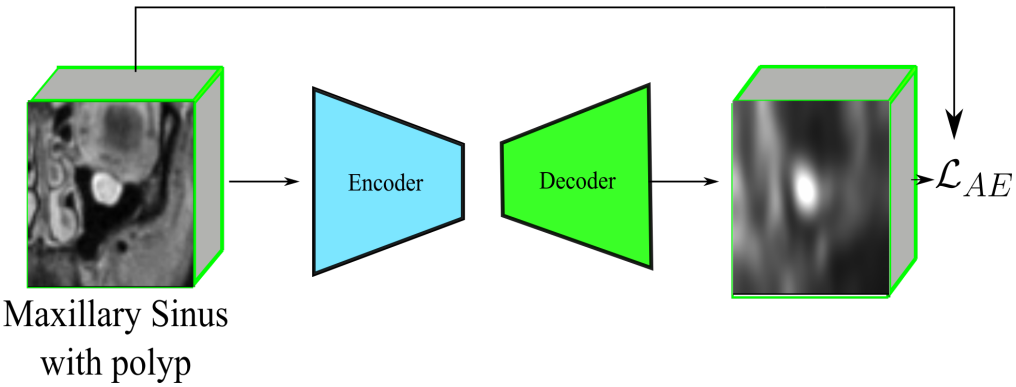

Fig. S1. Illustration of Autoencoder: The Autoencoder compresses the Maxillary Sinus (MS) volume in a nonlinear manner, creating a latent representation. It subsequently decompresses this representation to reconstruct the original MS volume. This process enables the encoder to learn valuable representations from the unlabeled dataset.

Formally,

[0]For correspondence send email to: debayan.bhattacharya@tuhh.de

$$\mathcal{L}_{AE} = |x - \hat{x}|$$

This constitutes our self-supervised training. After training, $D(.)$ is discarded, and $E(.)$ is fine-tuned, as explained in the main paper. Figure S1 illustrates the autoencoder.

2. DENOISING AUTOENCODER

The Denoising Autoencoder (DAE) closely resembles the autoencoder described earlier, with the key distinction that the encoder and decoder incorporate skip connections, and the input image is perturbed with Gaussian noise. Let $x' = x + \varepsilon$, where $\varepsilon \sim \mathcal{N}(\mu, \sigma^2)$. Once x is perturbed by Gaussian noise, it is processed through DAE, yielding $\hat{x} = D(E(x'))$. Training DAE involves the L1 loss, $|x - \hat{x}|$, applied to D_u. Skip connections can be used here because the added noise prevents the trivial copying task. After DAE training, $D(.)$ is discarded, and $E(.)$ is fine-tuned for the subsequent task.

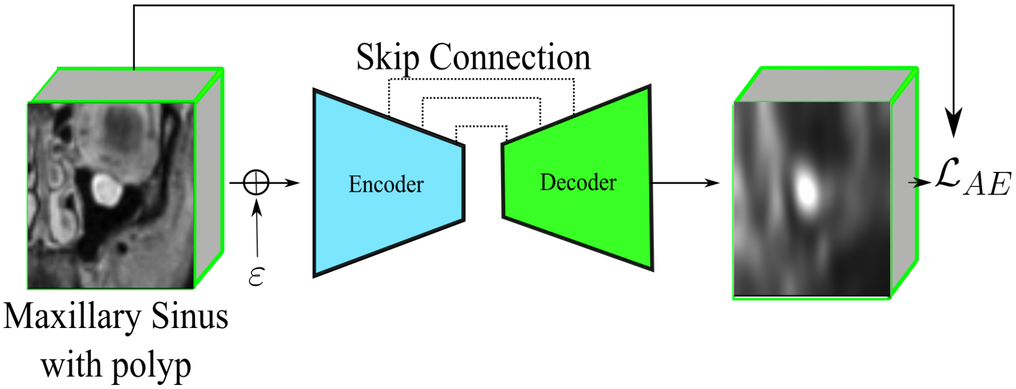

Fig. S2. Illustration of DAE: Gaussian noise is introduced into the MS volume, prompting the U-Net model to acquire denoising capabilities. As a result, the model not only removes the noise but also learns representations from the unlabeled data.

Figure S2 illustrates the DAE.

3. BOOTSTRAP YOUR OWN LATENT (BYOL)

In BYOL, self-distillation is employed to prevent collapse during training. Two networks, along with a predictor network, map one network's outputs to the other's. The network predicting the output is called the online (student) network, denoted as $E_s(.)$, while the network being predicted is called the target (teacher) network, $E_t(.)$. The predictor network is defined as $P(.)$.

The loss function is as follows:

$$\mathcal{L}_{\mathcal{BYOL}}(\theta_s, \theta_p) = \mathop{\mathbb{E}}_{(x,t_1,t_2 \sim (D_u, T_1, T_2)} [\|\text{renorm}(P(E_s(t_1(x))) - \text{renorm}(E_t(t_2(x))\|_2^2]$$

The vectors predicted by the student and teacher networks are L_2 normalized using:

$$\text{renorm}(v) = \frac{v}{\max(\|v\|_2 + \epsilon)}$$

where ϵ is set to 1×10^{-12}. E_s is parameterized by θ_s, and $P(.)$ is parameterized by θ_p. $x \sim D_u$ is the input sampled from the unlabeled dataset D_u. $t_1(x)$ and $t_2(x)$ represent two augmented views of x, with $t_1 \sim T_1$ and $t_2 \sim T_2$ denoting two data augmentations. The target network $E_t(.)$ shares the same architecture as $E_s(.)$ and is updated using exponential moving average (EMA), with ξ controlling the update speed:

$$\theta_t \leftarrow \xi \theta_t + (1 - \xi \theta_s$$

Following training, E_s is discarded, and only E_t is fine-tuned for the downstream task. Figure S3 illustrates BYOL.

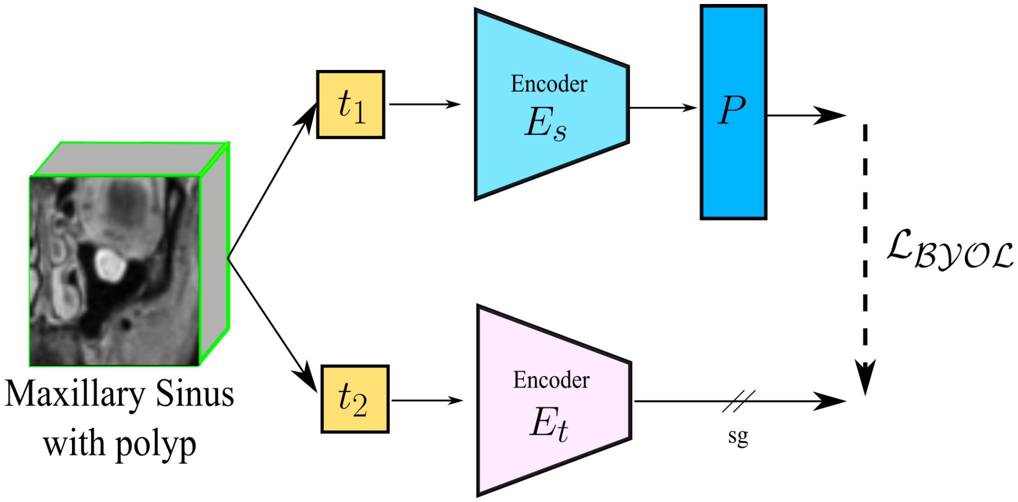

Fig. S3. Illustration of BYOL: The student and target encoders strive to generate similar representations for randomly augmented views of the same MS volume. This process, emphasizing similarity, encourages both encoders to learn transformation-invariant representations. The target encoder is updated by exponential moving average of the student encoder weights.

4. SIMSIAM

SimSiam simplifies BYOL by removing EMA. Consequently, the loss function is updated as:

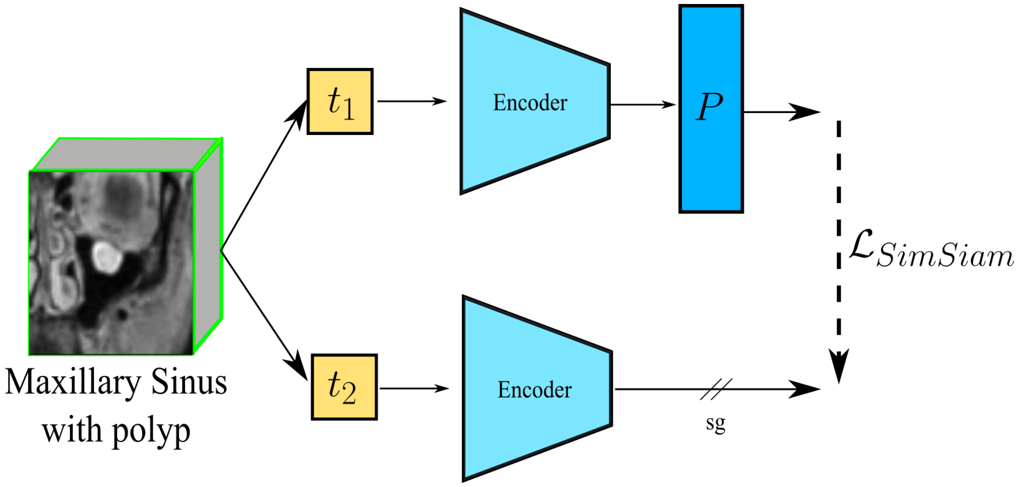

Fig. S4. Illustraion of SimSiam: Similar to BYOL except that the student and target encoders share weights. The encoder strives to generate similar representations for randomly augmented views of the same MS volume. This process, emphasizing similarity, encourages both encoders to learn transformation-invariant representations. SimSiam is BYOL without exponential moving average update of the teacher network.

$$\mathcal{L}_{SimSiam}(\theta_s, \theta_p) = \mathop{\mathbb{E}}_{(x,t_1,t_2 \sim (D_u, T_1, T_2)} [||\text{renorm}(P(E(t_1(x))) - \text{sg}(\text{renorm}(E(t_2(x))||_2^2]$$

Here, $E(.)$ is an encoder network. The two branches share $E(.)$, but one branch has the predictor network $P(.)$, making the branches asymmetric. Figure S4 illustrates SimSiam.

5. SIMCLR

SimCLR learns visual representations by promoting similarity between two augmented views of an image $x \in \mathbb{R}^{64\times64\times64}$. After encoding each view with the encoder $E(.)$, SimCLR utilizes

a projector network (often a multi-layer perceptron with ReLU activation) to map the initial embeddings into a transformed hyperspace. Contrastive loss encourages similar views from the same image to be close and dissimilar views from different images to be separated in the hyperspace.

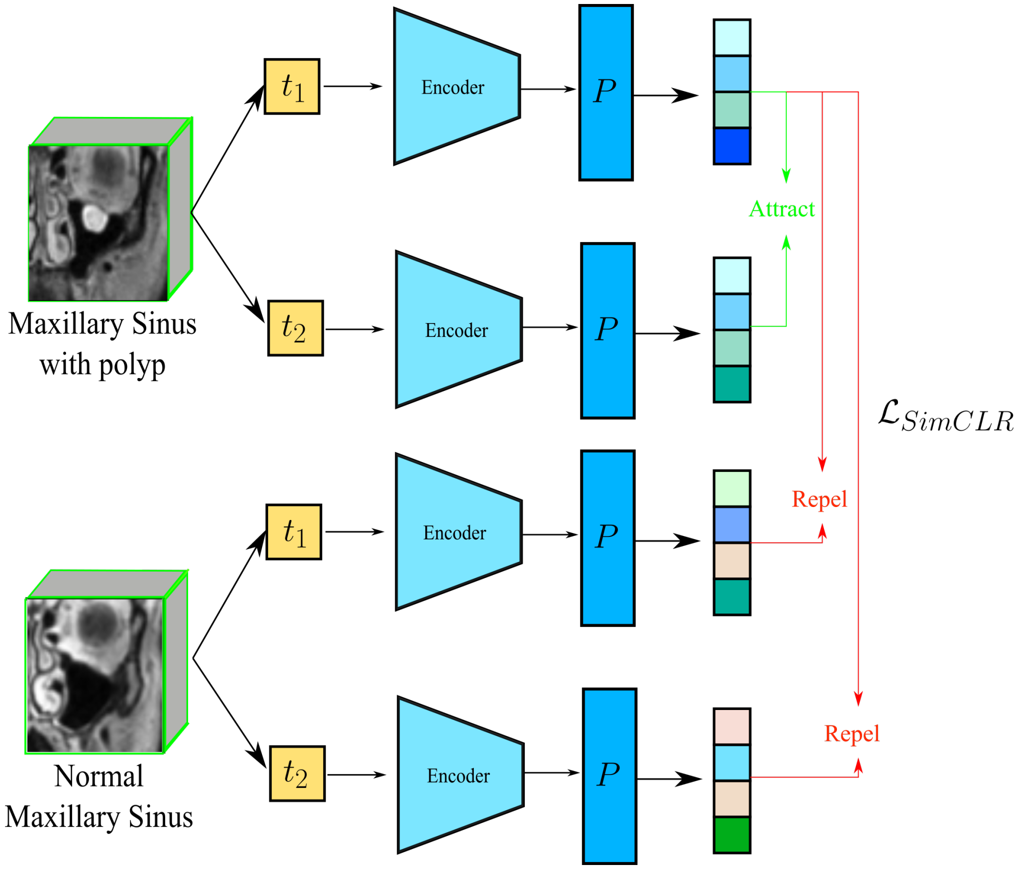

Fig. S5. Illustration of SimCLR: The encoder aims to generate similar representations for augmented versions of a single image while also working to ensure dissimilarity between augmented versions derived from different images.

Formally:

$$\mathcal{L}_{SimCLR} = -\log \frac{e^{\mathrm{sim}(z_i,z_j)/\tau}}{\sum_{k=1}^{N} e^{\mathrm{sim}(z_i,z_k)/\tau}}$$

where $z_i = \mathrm{renorm}(P(E(t_i(x))))$ and $z_j = \mathrm{renorm}(P(E(t_j(x))))$, and $P(.)$ is the prediction network. $t_i \sim T_i$ and $t_j \sim T_j$ denote two data augmentations. $sim(.)$ computes the cosine similarity between the two vectors, and N is the mini-batch size. After training, $E(.)$ is fine-tuned for the downstream task. Figure S5 illustrates SimCLR.

6. SPARSE AND HIERARCHICAL MASKED MODELING

Sparse masKed modeling with hierarchy (SparK) learns representations by masking images and inpainting them based on context provided by the unmasked image portions. This strategy of pretraining as proven to be very advantageous to Vision Transformers (ViT) but the same strategy is not directly applicable to CNNs due to their hierarchical architectural design. Unlike ViTs, which divide images into non-overlapping patches, CNNs operate on regular grids with overlapping windows. Simply dropping or masking patches in CNNs would result in information loss. Zeroing out masked pixels and feeding the resulting "mosaic" to a CNN would cause significant distribution shifts and other issues, making it an imperfect solution. In this method,

sparse convolution is utilized to prevent information leakage, and it can seamlessly integrate with any CNN without requiring backbone modifications. Sparse convolution operates efficiently by computing only at visible locations, addressing concerns like "pixel distribution shift" and "mask pattern vanishing." Figure S6 illustrates SparK training.

7. IMPLEMENTATION DETAILS

$E(.)$, $E_t(.)$, and $E_s(.)$ are 3D variants of the ResNet18 architecture. $E'(.)$ is a 3D variant of the ResNet18 architecture containing sparse convolutional operations. Decoder $D(.)$ used in SparK is a lightweight decoder containing 3 3D transpose convolutional blocks. Model optimization employs Layer-wise Adaptive Rate Scaling (LARS) with a learning rate of 0.2. Pretraining includes 500 epochs with a linear warmup of 20 epochs followed by cosine annealing with a batch size of 256. The model weights with the lowest validation losses are saved for both the self-supervision and fine-tuning stages. Various data augmentation techniques are applied, including random affine transformations, random flipping of the coronal, axial, or sagittal planes, and random Gaussian noise, each with a 50% probability. For DAE, $\mu = 0$ and $\sigma = 0.6$. $\xi = 0.99$ is used for BYOL. The prediction network $P(.)$ is an MLP with input and output dimensions of 512, with batch normalization layers and ReLU activation between layers for BYOL, SimSiam and SimCLR. The feature vectors are 512-dimensional for BYOL, SimSiam, and SimCLR. For SparK training, we have adapted the code [1] to accomodate 3D images.

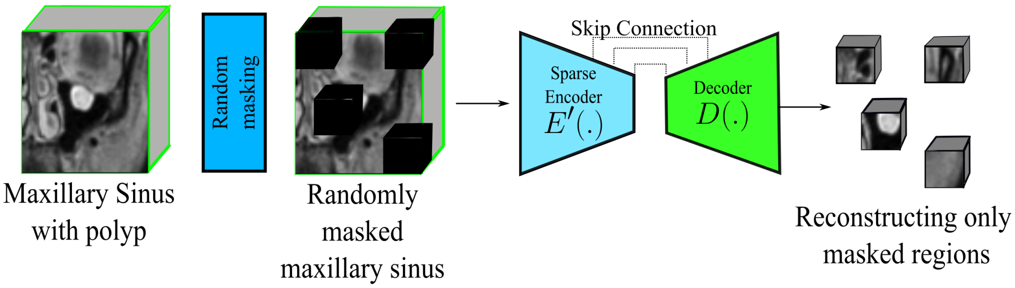

Fig. S6. Illustration of SparK: The sparse encoder and decoder inpaints only the randomly-masked regions of the input image to learn effective representations.

8. EFFECT OF LOSS FUNCTION ON THE SELF-SUPERVISION TASK

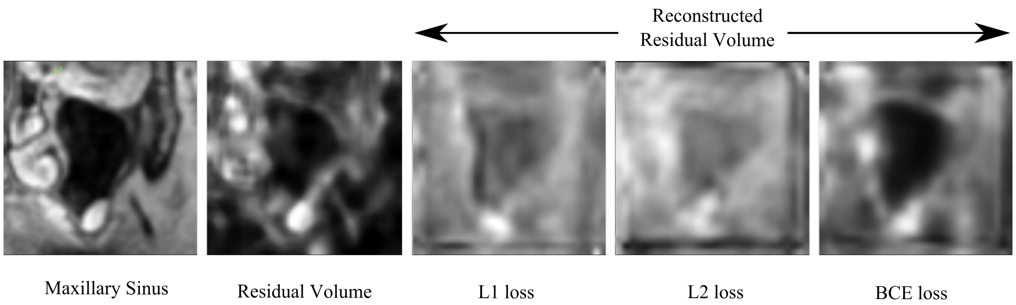

Fig. S7. Effect of reconstruction loss on the self-supervision task. Reconstructed volume of CNN trained with L1 and L2 loss generate a blurred version of the residual volume whereas the CNN trained with BCE loss reconstructs better residual volumes.

The choice of a loss function holds significant importance in reconstruction tasks. Achieving a more accurate reconstruction of the residual volumes may directly influences the quality of the representations learned. To comprehensively investigate this aspect, we conducted an analysis of the influence of different reconstruction loss functions L_{recon} on the self-supervision task and assessed their impact on the downstream task. This analysis is based on the performance of $E(.)$

[1] https://github.com/keyu-tian/SparK

Table S1. Performance metrics with 95% confidence interval for different loss functions on the self-supervision task

Loss function	AUROC	AUPRC	F1
L1	0.75 (0.67-0.82	0.71 (0.63-0.79	0.62 (0.54-0.71)
L2	0.79 (0.75-0.84	0.75 (0.68-0.81	0.64 (0.52-0.75)
BCE	**0.81 (0.74-0.88)**	**0.79 (0.71-0.87)**	**0.67 (0.58-0.77)**

pretrained to reconstruct residual volume using L1, L2 or BCE loss function and fine-tuned using 10% of D_l.

All our MS volumes and residual volumes are normalised to a range of 0 and 1. We post-process the residual volume by median filtering with a kernel size of 5. Our findings, as demonstrated in Table S1, reveal that employing the BCE loss leads to an improvement of 2.53%, 5.33% and 4.68% in terms of AUROC, AUPRC and F1, respectively, relative to the second-best performing model pretrained using the L2 loss function. These results establish BCE loss as the most beneficial in the self-supervision task, facilitating the learning of more transferable features for our downstream classification task.

To visually demonstrate the effect of the reconstruction loss, Figure S7 provides a qualitative representation of the reconstructed residual volumes generated by the CNN using different loss functions.

Our experiments revealed that employing BCE loss in the self-supervision task function resulted in the best reconstructed residual volumes. We attribute this improvement to the nature of BCE loss, which effectively emphasizes class separation and handles sparse data and outliers—characteristics crucial for our crude segmentation task. Furthermore, considering the segmentation nature of our self-supervision task, it is essential to choose an appropriate loss function that aligns well with this objective. In this regard, BCE loss emerges as a fundamentally superior choice for our task. In contrast, L1 and L2 loss treat each pixel equally, potentially undermining class separability, and L2 loss is particularly susceptible to the influence of outliers, leading to sub-optimal optimization. Our analysis regarding the impact of the CAE training set size has demonstrated that the inclusion of a substantial cohort of normal MS volumes yields notable benefits for both the self-supervision task and the subsequent downstream task.

9. ANALYSIS OF POST-PROCESSING ON THE SELF-SUPERVISION TASK

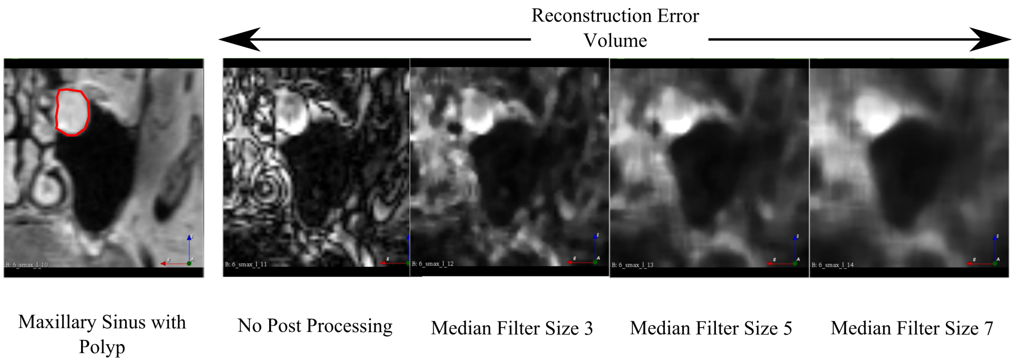

Fig. S8. Effect of median filtering on the reconstruction error volume. The polyp in the maxillary sinus is marked with red contours.

In this section, we investigate the impact of post-processing on the representations learned during the reconstruction task. To achieve this, we generate a residual volume by subtracting the output volume of the CAE from the input MS volume. We then employ median filtering to smooth out small reconstruction errors that may be considered outliers, with the kernel size of the median filter controlling the extent of smoothing. Median filtering retains larger reconstruction errors which may provide more meaningful information regarding the anomaly. Our hypothesis

Table S2. Performance metrics with 95% confidence interval with respect to the median filter kernel size

Median Filter Size	AUROC	AUPRC	F1
0	0.78 (0.73-0.83	0.74 (0.70-0.79	0.66 (0.59-0.72)
3	0.78 (0.71-0.83	0.74 (0.67-0.80	0.63 (0.54-0.73)
5	**0.81 (0.74-0.88)**	**0.79 (0.71-0.87)**	**0.67 (0.58-0.77)**
7	0.79 (0.72-0.85)	0.75 (0.68-0.81	0.65 (0.58-0.71)

is that the smoothed residual volume may have an influence on the representations learned in the self-supervision task. An ideal residual volume would have high reconstruction error localized within the anomaly mass and no reconstruction error on the unaffected regions of the MS or in other words, a segmentation mask with 1 for anomaly mass and 0 otherwise. Figure S8 shows the effect of median filters of different sizes on the reconstruction error volume. Median filter of size 3 and beyond suppresses the reconstruction errors localised on the nasal area.

Our objective is to understand how varying kernel sizes of the median filter affect the quality of representations learned by a CNN. The CNN is initially trained using BCE loss to reconstruct the residual volumes that have been subjected to different post-processing techniques. Subsequently, we perform finetuning on the CNN using 10% of the D_l initialized with weights obtained from the self-supervision task. It is important to note that the impact of the median filter kernel size on learned representations during the self-supervision task can be confounded by the potentially improved quality resulting from larger finetuning training set sizes. The results, presented in Table S2, indicate that a filter size of 5 achieves the highest AUPRC, AUROC and F1 scores, suggesting that a kernel size of 5 is optimal for addressing our specific problem statement. Applying a median filter with a 5-sized kernel improved our results by removing outliers while preserving important reconstruction errors in anomalies. These findings underscore the significance of post-processing techniques in enhancing the quality of representations learnt from the unlabelled dataset.

10. EFFECT OF MASKING RATIO AND PATCH SIZE FOR MAE PRETRAINING USING SPARK

We experimented with patch size (PS) $8 \times 8 \times 8$ and $16 \times 16 \times 16$. For each of these patch sizes, we varied the masking ratio (MR) by randomly masking the input images at 60%, 75% and 90%. Of all the PS and MR variations, we chose the CNN with PS and MR that had the highest AUPRC when finetuned on 10% labelled training dataset. PS 8 with MR of 60% had the highest AUPRC at 0.754 followed by PS 16 with MR 60% at 0.748. We used PS 8 and MR 60% as the SparK state-of-the-art in our main manuscript. Table S3 shows the classification performance using different pretraining configurations. Figure S9 shows the bar chart of the various SparK configurations.

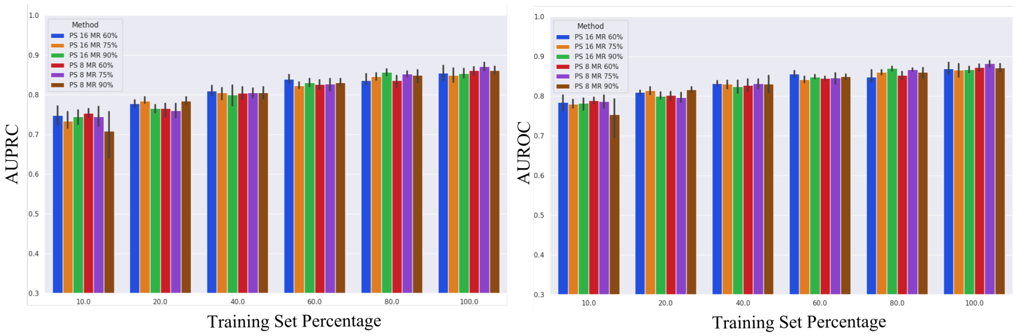

Fig. S9. (LEFT) AUPRC trend vs training set percentage (RIGHT) AUROC trend vs training set percentage for different patch size (PS) and masking ratio (MR) for MAE trained CNN using SparK.

Table S3. Mean and 95% confidence intervals of the different metrics for different MAE pre-training of 3D ResNet18 using SparK framework

Patch Size	Mask Ratio %	Training Set Percentage D_l	AUPRC	AUROC	F1
16	60	10	0.748 (0.711, 0.785)	0.785 (0.754, 0.816)	0.660 (0.632, 0.688)
16	60	20	0.778 (0.764, 0.792)	0.810 (0.803, 0.818)	0.687 (0.673, 0.701)
16	60	40	0.810 (0.792, 0.829)	0.833 (0.823, 0.842)	0.710 (0.697, 0.722)
16	60	60	0.840 (0.823, 0.857)	0.856 (0.843, 0.870)	0.733 (0.720, 0.745)
16	60	80	0.836 (0.813, 0.859)	0.848 (0.823, 0.874)	0.717 (0.682, 0.752)
16	60	100	0.855 (0.825, 0.885)	0.869 (0.847, 0.892)	0.752 (0.718, 0.785)
16	75	10	0.735 (0.703, 0.767)	0.780 (0.763, 0.797)	0.644 (0.622, 0.665)
16	75	20	0.785 (0.772, 0.799)	0.814 (0.799, 0.829)	0.691 (0.672, 0.711)
16	75	40	0.806 (0.780, 0.832)	0.831 (0.815, 0.848)	0.710 (0.690, 0.730)
16	75	60	0.824 (0.811, 0.836)	0.842 (0.830, 0.855)	0.708 (0.685, 0.732)
16	75	80	0.847 (0.832, 0.861)	0.860 (0.850, 0.871)	0.733 (0.716, 0.750)
16	75	100	0.849 (0.821, 0.877)	0.866 (0.839, 0.892)	0.746 (0.693, 0.799)
16	90	10	0.745 (0.717, 0.773)	0.783 (0.758, 0.808)	0.654 (0.632, 0.676)
16	90	20	0.766 (0.749, 0.782)	0.801 (0.787, 0.814)	0.681 (0.668, 0.694)
16	90	40	0.800 (0.759, 0.841)	0.825 (0.798, 0.852)	0.692 (0.638, 0.745)
16	90	60	0.831 (0.815, 0.847)	0.848 (0.840, 0.856)	0.722 (0.695, 0.749)
16	90	80	0.858 (0.844, 0.871)	0.870 (0.861, 0.879)	0.745 (0.723, 0.768)
16	90	100	0.854 (0.835, 0.874)	0.867 (0.853, 0.880)	0.749 (0.722, 0.776)
8	60	10	0.754 (0.738, 0.769)	0.789 (0.777, 0.802)	0.656 (0.639, 0.673)
8	60	20	0.766 (0.739, 0.793)	0.802 (0.784, 0.821)	0.672 (0.655, 0.689)
8	60	40	0.804 (0.780, 0.829)	0.828 (0.804, 0.852)	0.708 (0.691, 0.726)
8	60	60	0.827 (0.809, 0.845)	0.846 (0.839, 0.853)	0.718 (0.696, 0.739)
8	60	80	0.836 (0.813, 0.859)	0.853 (0.837, 0.868)	0.731 (0.716, 0.746)
8	60	100	0.862 (0.843, 0.880)	0.872 (0.858, 0.887)	0.755 (0.740, 0.770)
8	75	10	0.746 (0.705, 0.786)	0.786 (0.761, 0.812)	0.662 (0.643, 0.682)
8	75	20	0.760 (0.729, 0.792)	0.797 (0.777, 0.816)	0.652 (0.578, 0.726)
8	75	40	0.806 (0.788, 0.824)	0.832 (0.813, 0.851)	0.707 (0.691, 0.723)
8	75	60	0.828 (0.802, 0.853)	0.847 (0.824, 0.869)	0.722 (0.693, 0.750)
8	75	80	0.853 (0.843, 0.864)	0.867 (0.862, 0.873)	0.745 (0.731, 0.760)
8	75	100	0.872 (0.856, 0.888)	0.882 (0.870, 0.895)	0.771 (0.750, 0.793)
8	90	10	0.709 (0.609, 0.809)	0.754 (0.671, 0.838)	0.603 (0.513, 0.693)
8	90	20	0.785 (0.771, 0.799)	0.817 (0.807, 0.827)	0.683 (0.670, 0.697)
8	90	40	0.805 (0.619, 0.992)	0.831 (0.561, 1.100)	0.707 (0.352, 1.061)
8	90	60	0.830 (0.817, 0.844)	0.849 (0.839, 0.859)	0.721 (0.703, 0.739)
8	90	80	0.850 (0.823, 0.877)	0.860 (0.840, 0.880)	0.741 (0.721, 0.761)
8	90	100	0.862 (0.846, 0.877)	0.872 (0.857, 0.887)	0.755 (0.738, 0.773)
